# An Automated “Hands-Off” Method for Sampling Mainstream Smoke from Cannabis Cigarettes

**DOI:** 10.3390/toxics12050313

**Published:** 2024-04-26

**Authors:** David E. Campbell, Chiranjivi Bhattarai, Yeongkwon Son, Andrey Khlystov

**Affiliations:** Organic Analytical Laboratory, Division of Atmospheric Sciences, Desert Research Institute, Reno, NV 89512, USA; dave.campbell@dri.edu (D.E.C.); chiranjivi.bhattarai@dri.edu (C.B.); yeongkwon.son@dri.edu (Y.S.)

**Keywords:** cannabis, marijuana, hemp, puff topography, chemical emission, risk

## Abstract

A simple-to-use, portable, and relatively inexpensive system for characterizing the chemical components of mainstream smoke from cannabis cigarettes was developed and tested by using commercial hemp cigarettes. The system is described, and its performance for reproducing actual user puff topographies is shown along with extensive chemical analysis data, including PAHs, carbonyls, and organic and elemental carbon, for a small set of initial samples. By using a solid-state flow meter and fast-response mass flow controller, the prototype can reproduce measured puff topography with excellent fidelity, which will allow users to accurately reproduce the actual inhalation patterns for various types of smoking products and consumers, and to collect samples of mainstream smoke without the need to bring test subjects or controlled substances into a laboratory.

## 1. Introduction

Cannabis represents a group of plants containing naturally occurring cannabinoids such as cannabidiol (CBD) and delta-9-tetrahydrocannabinol (THC). THC is the compound that produces a “high” sensation in users. Hemp is a legal term for *Cannabis sativa* containing less than 0.3% THC by dry weight. Marijuana is a type of cannabis plant containing high THC (3–30% dry weight) and is a psychoactive drug regulated as a Schedule I controlled substance by the US federal agency [1]. The recent legalization of cannabis and cannabis-derived products for medical use in most of the United States, as well as for recreational marijuana use in many states, has resulted in the widespread production and use of commercially produced inhalable cannabis products [2].

This rapid growth in legal cannabis sales has not been accompanied by increased research into the health effects of long-term use of these products, due in part to severe restrictions on access to marijuana faced by laboratories receiving federal funding, despite increasing evidence of potentially serious health effects [3,4]. University of Mississippi under contract with the National Institute on Drug Abuse (NIDA) is the only research facility in the US licensed by the federal government to produce research marijuana, and access to their output is tightly controlled and generally limited to concentrated extracts of THC rather than the dried marijuana flowers smoked by most users. Although state-legal commercial cannabis products may be subjected to chemical analysis to determine human health risk, potency, and screen for pesticide residues and the National Institutes of Health (NIH) has supported research focused on specific cannabinoids, there has been very little analysis of the myriad of compounds that are inhaled by users when those products are smoked. The methods and instruments commonly used for inhalation studies of tobacco products have been applied to cannabis by a few research organizations [5,6,7], but the high cost (many thousands of US dollars) and the complexity of those methods limits the scope to a few anecdotal cases that cannot represent the wide array of products available to the public. 

As millions of people continue to use legal marijuana products, this lack of information on harmful compounds inhaled could lead to a public health crisis similar to the epidemic of lung cancers that resulted from tobacco cigarettes [8]. The need for such research was recently illustrated by the Center for Disease Control and Prevention (CDC), which issued a warning against use of electronic “vaping” products, particularly those containing THC, after serious respiratory conditions and several deaths of the users were reported in more than 25 states [9]. Also, recent research has demonstrated that when otherwise benign liquid flavorings are subjected to rapid heating in e-cigarettes, they can produce potentially hazardous by-products [10,11]. Although specific cannabis extracts like THC and CBD have been studied extensively, there are very few quality data available on the chemical composition of aerosol produced by combustion or evaporation of cannabis plant material. Access to consistent, detailed chemical analysis of the actual gaseous and particulate compounds that are inhaled by users of commercial marijuana products would be of great value to public health agencies and the general public. 

In order to address this need, we constructed a portable, low-cost system for collecting mainstream smoke from cannabis-related products (i.e., combustible or vaporized marijuana and hemp products). The sampling system can collect real-world use profiles of puff topography for cannabis-related products and mimic those profiles to collect smoke samples equivalent to those inhaled by users. To demonstrate its operation, we performed a limited set of experiments in which representative chemical compounds were measured in the mainstream smoke of a commercial hemp cigarette. The simple user interface and low-cost design of this system will allow it to be deployed and operated outside of a laboratory environment and without requiring the presence of a trained technician, facilitating the collection of emissions samples from actual cannabis product users.

## 2. Materials and Methods

### 2.1. Cannabis Smoke Sampling System Design

**Figure 1:**
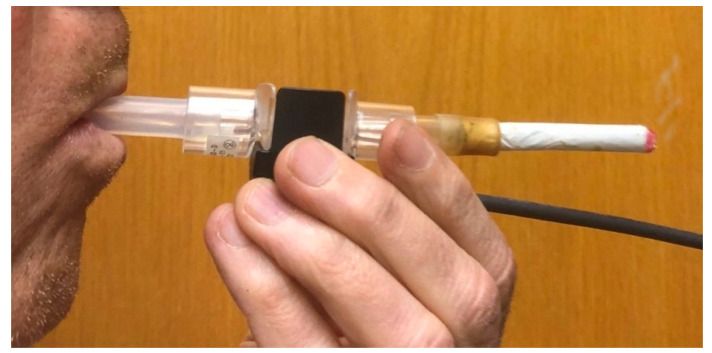
Photo of flow meter for puff profile characterization in use.

**Figure 2:**
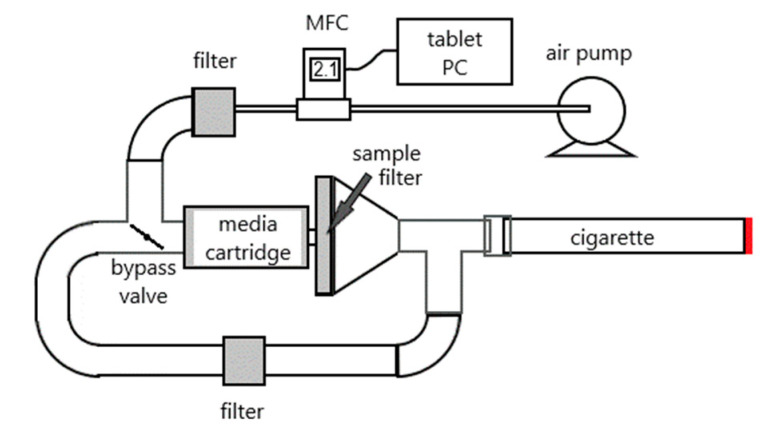
Schematic of portable cigarette smoke sampler.

Our “puff topography device” consists of a small, inexpensive flow meter (SFM3400; Sensirion, IL, USA) that uses a solid-state sensor to measure the air flow rate through it with high accuracy, fast-time resolution, and very low resistance. Since this meter is designed to measure the inspiration rate of medical patients, it was relatively easy to adapt it to our purpose by connecting a cigarette to the inlet side and drawing air through the cigarette and flow meter by inhaling from the outlet side (Figure 1). Short lengths of food-grade silicone tubing were used at the inlet and outlet of the flow meter to avoid direct contact and adapt the diameter to that of a cigarette. More sophisticated mouth pieces and vaping device adapters could be used if needed. The sensor is also relatively inexpensive (~USD 40 as of the time of this publication) and thus can be replaced frequently. A version of the flow meter used in our instrument that is specifically designed for ventilation or anesthesia devices, allowing autoclaving or cleaning with medical agents for sterilization and disinfection without loss of sensor functionality, is also available. Cannabis smoke deposits, such as tar, can be cleaned by using a cleaning agent. For further quality assurance checks, the output signal from the mass flow controller in the smoking machine (see Figure 2) can be used as a flow rate reference to verify sensor performance. 

The air flow rate is measured and recorded each millisecond by using a tablet PC and software supplied by the meter manufacturer. A Python program was written to read the recorded data, identify the individual puffs, and characterize them in terms of total volume, maximum flow rate, and duration (Supplementary Material S6). Air flow data for each puff were averaged to 100 msec intervals and saved in a simple delimited text file for use as input to control the smoking machine, thereby realistically simulating actual smoking behavior. This is important since the actual emissions from inhalable tobacco- and cannabis-related products may vary with puff topography. In particular, the wide range of smokable and vaping products available to cannabis users combined with differences in user intent (e.g., medical vs. recreational) are likely to result in a broad range of smoking behavior that cannot be accurately represented by any “standard” puff topography.

A “portable smoking machine” was assembled by using a fast-response mass flow controller (MFC) with digital signal remote control (Alicat MC series; Alicat, AZ, USA) connected to a tablet PC via USB serial port (Figure 2). The MFC was integrated into a sampling train that allowed the emissions from a test cigarette to be drawn through a filter to capture the smoke particles or routed through a bypass channel when lighting the cigarette or not running the puff sequence. If desired, a cartridge containing material to capture volatile components of smoke (e.g., thermal desorption tubes or sorbent resin tubes) can be included behind the filter. A Python program allows the user to manually control the air flow rate or initiate a pre-selected sequence of puffs by touching virtual buttons on the tablet’s touchscreen. The total cost of materials for the prototype was approximately USD 2,300 (most of which was for the MFC), but that could be substantially reduced if multiple units were to be constructed by using OEM components. Including a solenoid bypass valve controlled by digital signal to a relay, which we were unable to implement due to supply-chain disruptions at the time, would increase the cost and complexity slightly but is recommended, as it would improve reproducibility for inexperienced operators.

### 2.2. Sampling System Evaluation

To test the device’s ability to measure and reproduce smoking topography, a puff profile was recorded and reproduced repeatedly. The accuracy of the reproduced profile was verified with an inline flow meter attached to the filter end of an unlit cigarette. Aerosol collection efficiency was tested by using a commercial hemp cigarette (Original Hempettes; Batch #: G1420-200; Wild Hemp, TX, USA). Hemp aerosols were generated by using the developed portable smoking machine under three puff topographies (low, medium, and high flows) representing the range of real-world cannabis users’ puff topography (Table 1) [12,13]. To provide some context for the chemical analysis of hemp smoke, samples from filtered tobacco cigarettes (1R6F reference cigarette) were also collected by using the Mass. Dept. of Public Health (MDPH) puff topography for medium–intensive users [13]. The hemp cigarette filters were removed so the samples would better represent the exposure experienced by marijuana smokers, who typically smoke material with minimal or no filtering. For chemical analysis, 3 puffs under each puff profile from a single hemp cigarette were collected on a single set of media, and replicate samples were collected for each puff profile. Analytical results from the replicate pairs of samples were combined so that they represented the sum of 6 puffs for each profile. This protocol was selected to produce sufficient material for accurate chemical analysis without clogging the filters or resulting in excessive saturation of the DNPH cartridges. Coincidentally, the unfiltered Hempettes burned faster than tobacco, so we were only able to collect about 6 puffs from each cigarette. A separate set of sampling media, consisting of 47 mm pre-fired quartz filters (QAT2500-VP; Pall Sciences, Ann Arbor, MI, USA) followed by DNPH cartridges (Sep-Pak DNPH-Silica cartridge; Waters, MA, USA) for bulk organic and elemental carbon, vapor-phase aldehydes, and cannabinoids, was collected in this manner for each profile shown in Table 1. In addition, samples for each of the 3 cannabis user puff topographies were collected onto 47 mm Teflon-impregnated glass fiber filters (TIGFs; Pall Life Sciences, Ann Arbor, MI, USA) for determination of polycyclic aromatic hydrocarbons (PAHs) and nitro-PAHs (NPAHs). The sampling media were immediately removed to cold storage for subsequent analysis at the DRI laboratories. Chemical analysis methods are described in Supplementary Material [10,11,14,15,16,17].

## 3. Results

### 3.1. Puff Topography Profile

**Figure 3:**
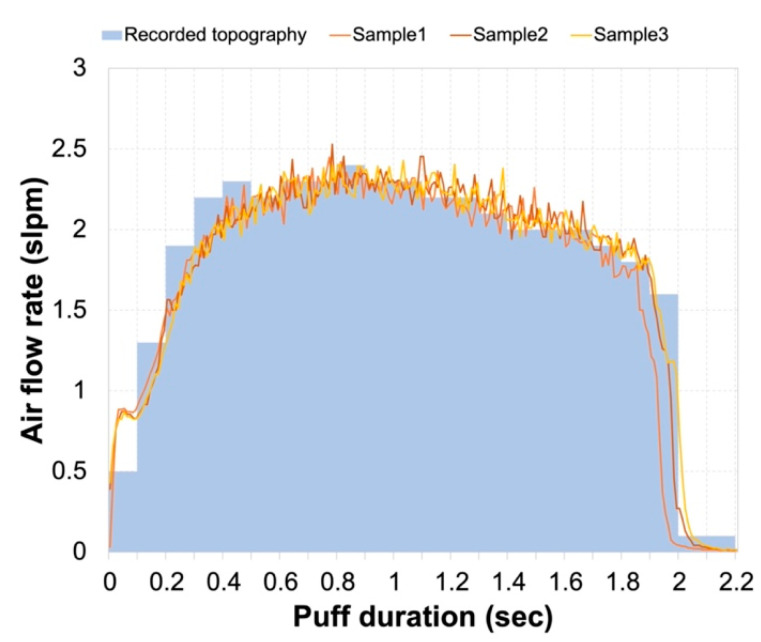
Comparison of recorded puff topography (sky blue-shaded area) and actual puff duration during sample collection (lines).

The portable smoking machine can accurately replicate the pattern of puff profiles with <100 ms time resolution (Figure 3, sky blue-shaded area). The total sample volume for the sequence of three puffs (line plots in Figure 3) was equal to the programmed puff volume within 5%. The average deviation from the recorded profile was 9% per 100 ms average, with a maximum deviation of 53%. More frequent sampling (e.g., 10 ms) is possible and was attempted in this study but did not prove useful due to the lag time of the serial port response. A more powerful PC or a dedicated micro-controller could potentially provide measurements with higher time resolution, resulting in a closer reproduction of the puff profile. Initially, we intended to use a Raspberry Pi or similar single-board computer as the controller and data-logger for the system but were unable to establish direct communication with the Sensirion flow meter. The manufacturer’s documentation suggests this is possible, but they did not respond to our requests for assistance with implementation.

### 3.2. Chemical Emission Profile

**Figure 4:**
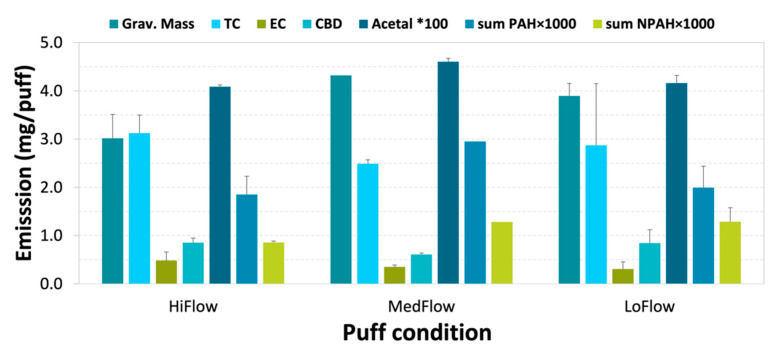
Summary of gravimetric mass (Grav. Mass), carbon analysis (total carbon [TC] and elemental carbon [EC]), and chemical analysis (cannabidiol [CBD], acetaldehyde×100 [Acetal × 100], sum of PAH compounds [sum PAH × 1000], and sum of nitro-PAH compounds [sum NPAH × 1000]) of mainstream hemp smoke under 3 different puff conditions (Hi, Med, and Low flows in Table 1). Concentrations shown are averages of replicate samples, and error bars indicate variation within pairs.

**Figure 5:**
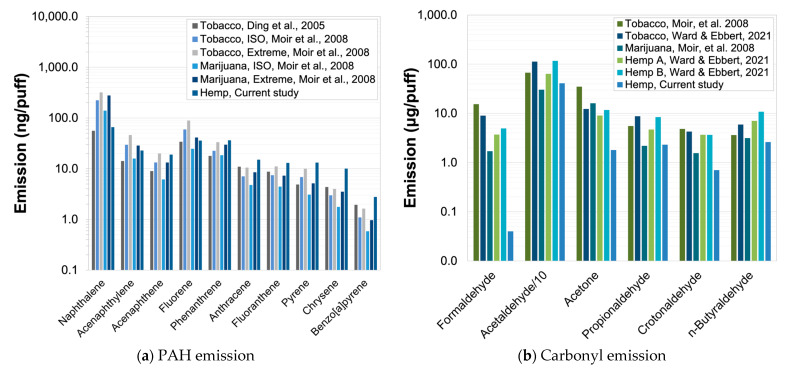
Comparison of measured amounts of (**a**) particle-phase polycyclic aromatic hydrocarbons (PAHs) and (**b**) carbonyls in unfiltered hemp from current study (Hemp, Current study) and mainstream tobacco, marijuana, or hemp smoke from the literature [7,18,19].

We were able to collect detailed chemical profiles that included the mass concentration of 75 PAH species, 35 nitro-PAHs, 14 aldehydes, 16 cannabinoids, total organic carbon (OC) and total elemental carbon (EC), and total particulate mass. The analytical results are summarized in Figure 4 and Figure 5, and the complete data set is included in the Supplementary Material (Table S1). Gravimetric mass concentrations shown in the figure were determined by weighing the TIGF filters before and after sampling. Total carbon and elemental carbon are derived from thermal–optical transmittance analysis of the quartz filters [17]. Carbonyl compounds, from the HPLC analysis of the DNPH cartridges [11], are represented in the chart by the concentration of acetaldehyde, which accounted for 80% of the identified species. The concentrations of PAHs shown in the chart are the sum of all compounds identified from the analysis of the TIGF extracts [15].

## 4. Discussion

This study developed a portable smoking machine that can easily collect and mimic cannabis users’ puff topography. Various sample collection media could be attached to the machine to collect mainstream chemical emissions. The machine could be operated by users; thus, researchers could collect valuable data without dealing with the Schedule I controlled substance regulation. 

Another objective of this study was to test if the sampling method would be useful to determine the significance of smoking behavior (i.e., puff topography) on emissions of the various chemical compounds. The initial data are inconclusive, since the variation in emission rates for a specific puff topography was similar to or greater than that between different topographies, as shown by the error bars in Figure 4. This may have been due to an inconsistent combustion rate of the hemp cigarette filler materials, which contain a mixture of the complete “aerial parts” of the plant according to the manufacturer. Determining the significance of puff topography and variability in emissions due to factors such as material source, processing, and rolling paper content and style will require a more extensive measurement program that is beyond the scope of this pilot project. 

To put the magnitude of the chemical analysis results in context, we compared the measured concentrations of organic and elemental carbon to those from filtered reference tobacco cigarettes by using the same puff profile. For the two pairs of samples we analyzed, the unfiltered hemp smoke contained 11.6 ± 0.8 times more organic carbon and 21.6 ± 7.1 times more elemental carbon than the reference tobacco cigarette. Although we did not collect samples from the tobacco cigarettes for PAH analysis, a comparison to published data for several types of unfiltered tobacco [18] indicates that the emissions are comparable, as shown in Figure 5a for the high-flow topography condition. We also compared our chemical analysis results to the limited number of similar data for cannabis that have been published [7,18,19]. As shown in Figure 5a,b, we measured similar or higher concentrations of PAHs, except naphthalene. Carbonyl compounds were generally much lower than other published values, except for acetaldehyde, which was one-third to two-thirds of the reported values. No comparable data for the other analytes we measured were available. It should be noted that due to the small number of samples collected and the use of only a single type of hemp cigarette, the chemical analyses presented here are not intended to be representative of the chemical content of mainstream smoke inhaled by all cannabis users. A more comprehensive characterization by using the research cigarette and other alternative products while intercomparing with commercial smoking machines (which, it should be noted, could also suffer from sampling biases) is desirable and hopefully can be conducted in the future but was outside the scope of our study.

To compare risks associated with tobacco and cannabis (i.e., marijuana and hemp) smoking, we estimated hazard quotients (HQs) for non-cancer risk and lifetime cancer risk (CR). Among the measured chemical compounds in this study and the literature, formaldehyde, acetaldehyde, and benzo[a]pyrene (BaP) were selected for risk estimation because they are the most hazardous compounds based on a previous tobacco smoke risk study, contributing more than half of the risk associated with hazardous and potentially hazardous compounds in cigarette smoke [20]. The observed concentrations from this current study and literature values [7,18,19] were combined to obtain emitted average formaldehyde, acetaldehyde, and BaP concentrations from tobacco, marijuana, and hemp (μg/m^3^). The reference concentration (RfC; μg/m^3^) and cancer unit risk [(μg/m^3^)^−1^] were obtained from the EPA CompTox Version 2.3 database. Exposure duration and average lifetime were 40 years and 78 years, respectively, for cancer risk estimation. The estimated HQs for tobacco, marijuana, and hemp were 0.300, 0.150, and 0.153, respectively, and CR values were 2.75 × 10^−6^, 9.97 × 10^−7^, and 1.15 × 10^−6^, respectively. Both HQs and CR values for tobacco were higher than those for marijuana and hemp. Considering that tobacco smokers use more cigarettes per day (9.3 ± 5.5 cigarette/day) [21] than marijuana users (2.4–4.5 joints/day) [22], tobacco smokers might face higher risks than cannabis users. It should be noted that the HQs and CR values estimated here were based on only three compounds among the various toxic chemicals emitted from the tested products. Additionally, more comprehensive studies are needed to better assess risks of hemp and cannabis product use. Different biological and psychoactive mechanisms of nicotine, THC, and CBD should also be considered in such future studies.

In summary, a practical method for characterizing the composition of mainstream smoke from cannabis products, which cannot be studied in house due to their federal classification as Schedule 1 drugs, was tested by using commercial hemp cigarettes as a surrogate product. Overall, this approach appears to have application as a means for laboratories and researchers to study potentially harmful components of smoke from cannabis products without running afoul of federal regulations and allowing them to evaluate a more representative range of products and usage styles. To better assess the health risks to the public, a wide range of products and behavior should be tested with sufficient scientific rigor to provide the necessary data for health impact studies and epidemiology. We hope that the method presented here can be a useful tool in that regard.

## Figures and Tables

**Table 1:** Hemp cigarette puff topographies used in sampling.

Parameter	Topography Condition			
	High Flow	Med Flow	Low Flow	MDPH
Puff duration (sec)	1.7	1.6	2.1	2.0
Average flow rate (LPM)	2.1	2.0	1.7	1.3
Max flow rate (LPM)	3.4	2.9	2.4	1.9
Puff volume (ml)	65.0	59.7	66.5	45.7
Inter-puff duration (sec)	30	30	30	30

## Data Availability

The data is provided in the Supplementary Material.

## Supplementary Materials

The following supporting information can be downloaded at: https://www.mdpi.com/article/10.3390/toxics12050313/s1, S1: Chemical analysis data, S2: PAH and NPAH analysis methods, S3: Carbonyl analysis method, S4: Cannabinoid analysis method, S5: Thermal/optical carbon analysis methods, S6: Cannabis topography/smoking machine parts and control programs, Table S1: Chemical compound emission from hemp and conventional cigarette under different puff profiles, Table S2: Cannabis topography/smoking machine part list. References [23,24] cited in Supplementary Materials.
