# Supplementary material for: An Automated “Hands-Off” Method for Sampling Mainstream Smoke from Cannabis Cigarettes
Source: Toxics. 2024 Apr 26;12(5):313. doi: 10.3390/toxics12050313 (PMC11125762; doi:10.3390/toxics12050313)
Supplement: Supplementary file 1 [file toxics-12-00313-s001.zip › HempPaper SI_final-review.pdf]

Supplementary Material

An Automated "Hands-off" Method for Sampling Mainstream Smoke from Cannabis Cigarettes

David E. Campbell, Chiranjivi Bhattarai, Yeongkwon Son, Andrey Khlystov
Organic Analytical Laboratory, Division of Atmospheric Sciences, Desert Research Institute, Reno, NV 89501, USA

S1. Chemical Analysis Data

Please see supplementary Table S1 file for detailed list of chemical compounds and their concentrations.

S2. PAHs and NPAHs Analysis Methods

Polycyclic aromatic hydrocarbons (PAHs) and nitrated-PAHs (NPAHs) were analyzed using gas chromatography mass spectrometry (GC/MS) method with electron ionization (EI) or chemical ionization (CI) for PAHs and NPAHs, respectively [1-3]. Hemp smoke was collected on pre-fired 47-mm diameter Teflon-impregnated glass fiber (TIGF) filters (47-mm in diameter, Fiber Film T60A20, Pall Life Sciences, Ann Arbor, MI, USA) for organic analysis. Deuterated internal standards (Sigma-Aldrich, St. Louis, MO, USA) were spiked onto the sample filters and extracted using an accelerated solvent extractor (ASE) instrument (ASE350, Thermo Fisher, Waltham, MA, USA). The ASE extraction parameters were temperature: 80 °C, solvents: dichloromethane followed by acetone (150 mL each), pressure: 10.3 MPa, and extraction time: 15 min. After extraction, the volume of extract solution was reduced to 1 mL under a gentle nitrogen stream, filtered with a 0.2-μm pore-size polytetrafluoroethylene membrane filter (Whatman, Florham Park, NJ, USA), and transferred into a 2-mL volume amber glass vial.

A Scion-456 GC, equipped with a CP-8400 autosampler and interfaced to EVOQ-TQ triple quadrupole Mass Spectrometer (Bruker, Billerica, MA, USA), was used to perform the PAHs and NPAHs analysis. PAHs were analyzed with electron impact (EI) gas chromatography mass spectrometry (GC-MS). Samples were injected using a splitless injections into a 5 % phenylmethylsilicone fused silica capillary column (DB-5MS, 30 m, Agilent Technologies, Palo Alto, CA, USA) with a 10-m length, integrated, deactivated guard column. NPAHs analysis was done separately using mild polarity high temperature fused silica capillary columns (DB-17ht,30 m, Agilent Technologies, Palo Alto, CA, USA) with a 10-m length, integrated, deactivated guard column with chemical ionization (CI) gas chromatography mass spectrometry (GC-MS). We used single ion monitoring (SIM) mode to improve the detection and quantification of the compounds. Based on checks with the 16 EPA PAHs, the limit of detection (LOD)

varied between 0.02 and 0.05 ng μL^{-1}. The limit of quantification (LOQ) for PAHs was calculated by multiplying the LOD by 3.3 (U. S. Food and Drug Administration/Center for Biologics Evaluation and Research, 1995) and thereby the LOQ for analyzed PAHs was in the range of 0.066 and 0.165 ng μL^{-1}.

S3. Carbonyl Analysis Method

Carbonyl compounds were analyzed using 2,4-Dinitrophenylhydrazine (DNPH) derivatization method [4, 5]. The DNPH cartridges (Sep-Pak XPoSure Plus Short Cartridge, WAT047205, Waters, Milford, MA, USA) were used to collect carbonyl compounds. After collection, cartridges were extracted with 2 mL of acetonitrile (HPLC grade, EMD Millipore, Burlington, MA, USA). A 2-μL aliquot was then injected into an HPLC system (Waters Arc HPLC, with Waters 2998 Photo Diode Array (PDA) Detector, Waters, Milford, MA, USA) equipped with a XBridge® BEH column (C18, 3.0 mm×75mm, 2.5μm, Waters, Milford, MA, USA). DNPH-carbonyl adducts were quantified at 360 nm, while full spectrum readings (210–400 nm) were used to confirm the identity of individual compounds. Carbonyl concentrations were quantified using eight-points (0.002 to 10 μg/mL) external calibration curves prepared from a certified calibration mixture (AccuStandard, New Haven, CT, USA). The weighting factor of $1/x^2$ is used to generate calibration curve and is acceptable with a $R^2 \geq 0.95$. The LOD for carbonyl varied between 0.002 and 0.024 μg/mL and corresponding LOQ for analyzed carbonyl was in the range of 0.008 and 0.081 μg/mL.

S4. Cannabinoid Analysis Method

Collected hemp smoke on TIGF filter were placed in a 15 ml centrifuge tube. 10 ml of methanol was added into the centrifuge tube and extracted for 1-hour using a sonicator. The procedure was duplicated, and two extracts were combined and concentrated under a pure nitrogen flow until 1 ml. The extracts were syringe filtered using a syringe filter (0.22 μm pore, 13 mm diameter, VWR, PA, USA) and transferred to an autosampler vial for HPLC/PDA analysis. The sample extracts (5 μl) were injected into Waters 2690 Alliance System with a model 996 photodiode array (PDA) detector equipped with CORTECS Shield RP18 column (2.7 μm, 4.6x150mm). Mobiles phases were (A) ultrapure water with 1% formic acid and (B) acetonitrile with 1% formic acid. The mobile phase gradient was 60% B at 0 min until 10 min, increase B to 70% until 32 min, increase B to 95% until 32.5 and hold for 2 min, then decrease B to 60% until 35 min and hold 3 min. Total runtime was 38 minutes at 1 ml/min flow rate.

S5. Thermal/Optical Carbon analysis Methods

In this method, a 0.5 cm^2 sample punch from a pre-fired 47mm diameter quartz filter is heated to 120, 250, 450 and 550°C in a pure helium atmosphere, then to combustion at temperatures of 550, 700 and 800°C in a 2% oxygen and 98% helium atmosphere. The carbon which evolves at each temperature is converted to methane and quantified with a flame ionization detector. The reflectance from the deposit side of the filter punch is monitored throughout the analysis. This reflectance usually decreases during volatilization in the helium atmosphere owing to the pyrolysis of organic material. When oxygen is added, the reflectance increases as the light-absorbing carbon is combusted and removed. Organic carbon is defined as that which evolves prior to re-attainment of the original reflectance, and elemental carbon is defined as that which evolves after the original reflectance has been attained. A more detailed description of the TOR/TOT carbon analysis procedure and its limitations can be found elsewhere [6-8]

S6. The cannabis topography/smoking machine parts and programs

Table S2. Cannabis topography/smoking machine part lists

**Component**	**Vendor**	**Model**	**Size**	**units**
Pump	GSS	Rocker 300	1/8 horse power	1
Flow meter	Sensirion	SFM 3400-D		1
MFC	Alicat	MC series	20SLPM	1
Barbed Inline filter	McMaster-Carr	8991T34	1/4" Tube ID	1
3-way valve	McMaster-Carr	4566K22	Brass Valve with Yor-Lok Fittings	1
Bypass filter holder	Advantec	662322	47-mm Polypropylene	1
Bypass filters	Whatman	1001-047	47mm diameter	1
Sample filter holder	BGI	4129	47 mm	1
Tubing adapters	McMaster-Carr	5182K274	1/4" Stem x 1/4" Sleeve SS	2
Tubing	Beduan Pneumatic		1/4" polyurethane	6’

Table S1 lists the parts used to build the cannabis topography/smoking machine that used in this manuscript. The flow meter and mass flow controller (MFC) measure topography or control puff topography to mimic the observed cannabis cigarette smoking topography. Other parts are filter and its holder, and tubes connecting cannabis cigarette, sampler, and vacuum pump.

The smoking machine control codes were written in Python. Below are program codes to record cannabis cigarette topography and to control puff profiles.

1. Program code for cannabis cigarette puff topography record

```
"""
Created on Mon Oct 26 11:44:19 2020
script to read smoking puff pattern from Sensirion flowmeter and reproduce it as output to MFC
@author: davec - Desert Research Institute
"""

import pandas as pd, numpy as np
import matplotlib.pyplot as plt

cpath = r"C:\myproject\pufftopo\\" # path to data directory (Windows)
# filename of raw puff flow patterns (recorded using Sensirion SFM3400 and software)
infile = cpath + r"\flowlog.csv"
# create output file to write results
outfile = infile.rstrip('.csv') + '.xlsx'
outcsv = infile.rstrip('.csv') + '_100ms.csv'
# Create a Pandas Excel writer using XlsxWriter as the engine.
Writer = pd.ExcelWriter(outfile, engine='xlsxwriter')

# import raw flow data to Pandas dataframe
df_import = pd.read_csv(infile, skiprows = 1, names = ['Time', 'flow'], nrows = 150000)
t0 = df_import.Time.iloc[0] # store initial time stamp
df_import.Time = df_import.Time - t0 # convert time stamp to elapsed seconds
df_import.to_excel(writer, sheet_name='rawData') # write the dataframe to excel sheet
df_import.to_csv(outcsv,index=False) # write the dataframe to text file

df = df_import.copy()
df[df < 0] = 0 # set negative values to zero
ndec = 1 # set number of decimals to round time stamps
df.Time = df.Time.round(ndec) # round ETs
df.rename(columns={'Time':'ET(s)'},inplace = True)
dfa = df.groupby(['ET(s)'], as_index=False).mean() # average flows by time stamp
```

```
dfa.flow = dfa.flow.round(1) # round flows to 0.1 lpm

# plot flow data
bp1 = plt.bar(dfa['ET(s)'],dfa['flow']) # make bar plot
plt.title('Puffs', fontsize=20)
plt.xlabel('ET (sec)')
plt.ylabel('lpm')
pname = cpath+'temp_chart.png'
plt.savefig(pname, bbox_inches='tight')
plt.show()

# give each puff a unique integer label
dfa['puffnum']=np.nan
pn = 0
for n in range(len(dfa)-1):
  if dfa.flow.iloc[n+1]>0:
    if dfa.flow.iloc[n]==0:
      pn = pn+1
    dfa.puffnum.iloc[n+1] = pn
# create table of puff parameters
npuffs = int(dfa.puffnum.max())
dpuffs = pd.DataFrame(columns=['seconds','avglpm','maxlpm','liters'])
for n in range(1,npuffs+1):
  dur = round(len(dfa[(dfa.puffnum == n)])/(10.000**ndec),1)
  if dur > 1: # skip peaks shorter than 1 sec
    avgf = round(dfa.flow[(dfa.puffnum == n)].mean(),2)
    maxf = round(dfa.flow[(dfa.puffnum == n)].max(),2)
    vol = round(avgf*dur/60,3)
    print ("vol = " + str(vol) + "    dur = " + str(dur))
    dpuffs = dpuffs.append(pd.Series([dur,avgf,maxf,vol],index=dpuffs.columns),ignore_index=True)
dpuffs.loc['avglpm'] = dpuffs.mean() # add row with mean parameters
dpuffs.to_excel(writer, sheet_name='puffparams') # write the dataframe to excel sheet

# plot all puffs on overlapping chart
```

```
for n in range(1,npuffs+1):
  l = 'puff'+str(n)
  dfn = dfa[dfa.puffnum == n]['flow'].reset_index(drop=True).rename(l) # select one puff record and
renumber ET
  dfn.index = dfn.index/(10.000**ndec)
  lp1 = plt.plot(dfn, label = l, marker='o') # create plot line for chart
  # create new table with separate column for each puff
  if n == 1:
    dfm = pd.DataFrame(dfn)
  else:
    dfm = dfm.join(dfn,how='outer')
dfm = dfm.fillna(0) # replace missing values with zero (some puffs may be longer than others)
dfm['avglpm'] = dfm.mean(axis=1) # calc average flow for each second
dfm['avglpm'].to_csv(cpath + 'puffavg.csv',header=True,index_label='ET(s)') # export mean flow per
second to csv

#create chart and save to file for export to output file
plt.legend()
plt.xlabel('ET (sec)')
plt.ylabel('lpm')
pname2 = cpath+'temp_chart2.png'
plt.savefig(pname2, bbox_inches='tight')
plt.show()

sname = str(1000/(10.000**ndec))+'msAvg' # generate string for worksheet label
dfa.to_csv(outcsv,index=False)
dfa.to_excel(writer, sheet_name=sname) # write the dataframe to excel sheet
writer.sheets[sname].insert_image('E2',pname) # add chart to sheet
writer.sheets[sname].insert_image('E22',pname2) # add chart to sheet

writer.close()
```

2. Program code for cannabis cigarette puff topography record

```
"""
Created on Nov 16 2023
script to send serial commands to Alicat MC mass flow controller (MFC) to simulate smoking topography
@author: davec - Desert Research Institute
"""

import serial, time, pandas as pd, tkinter, csv, sys
from os.path import exists
from datetime import datetime, timedelta
import winsound # package to enable audio signal from PC
MFCport = "COM8" # select com port to MFC (Windows)
RelayPort = "COM#" # select com port to solenoid valve relay (Windows)
baud = 19200 # set serial port baud rate
devID  = 'A' # set MFC device ID
operator = 'DC'

window = tkinter.Tk() # establish GUI popup window

def SendSerMFC(astr): # function to send conmmands to MFC
    #print(astr)
    serMFC.write(astr.encode())
    serMFC.write('A\r'.encode()) # poll MFC for dataframe

def ReadSerMFC(): # function to return data from MFC
    getData = serMFC.readline()
    setpt = str(getData).split()[5]
    print()
    print("New MFC setpoint = " + setpt)
    print()

def SetMFC(flpm): # function to read puff sequence and send to MFC
```

```
if flpm == 'S': # run sequence of flow settings to simulate puff
    print ()
    print ("Starting Puff sequence")
    # read puff sequence data
    cpath = r"C:\myproject\\" # path to data directory (Windows)
    infile = r"pufftopo\puffavg.csv" # location and name of file containing recorded puff topography
    df = pd.read_csv(cpath + infile, usecols=['ET(s)','flow']) # copy file to Pandas dataframe
    sampvol = round(df['flow'].sum()/600,3) # calculate total puff volume
    puffseq = df['flow'].tolist() # extract flow steps from dataframe to list
    # set MFC to initial flow rate for stabilization
    print()
    flpm = puffseq[0]
    print('Setting flow to '+ str(flpm) +' lpm')
    astr = devID + 'S' + str(flpm) + '\r'
    SendSerMFC(astr)
    time.sleep(2.0)
    winsound.Beep(2000, 250) # audible signal; parameters are hz, ms
    print ("Switch valve to sampling position")
    ti = datetime.now() # store start time to variable

    # if using remote controlled solenoid valve include following lines
    '''
    # switch bypass valve to sampling position
    try:
        serRelay = serial.Serial(RelayPort, baud, timeout = 1)
        print("Relay comm port " + RelayPort + ' open')
    except:
        print('unable to open Relay Comm Port ' + RelayPort)
        return
    serRelay.setDTR(True) # turn on pin 4 of DB9 serial connector
    '''

    # send sequence to MFC
    nreps = 1 # set number of times to run sequence
```

```
for j in range(nreps):
    print(datetime.now())
    for i in range(1,len(puffseq)):
        # adjust MFC setting to next level
        astr = devID + 'S' + str(puffseq[i]) + '\r'
        SendSerMFC(astr)
        print(datetime.now().strftime("%M:%S.%f")[:-2] + '  puff ' + str(j+1)
              + ' -step ' + str(i) + ': ' + str(puffseq[i]) + 'lpm') # display current time and sequence step
        # hold flow rate for specified interval (delay may need to be adjusted to match comm latency)
        t0 = datetime.now()
        tdelta = timedelta(milliseconds = 0)
        while tdelta < timedelta(milliseconds = 75):
          tdelta = datetime.now() - t0
          time.sleep(0.001)
    et = datetime.now() - ti
    # reset flow to zero
    astr = devID + 'S' + str(0) + '\r'
    SendSerMFC(astr)
print ()
print ('sequence of '+ str(nreps) + ' Puffs complete')
now = datetime.now()
dt_string = now.strftime("%Y-%m-%d_%H:%M:%S")
print("date and time =", dt_string)
print ('total sample volume = ' + str(sampvol) + ' liters')
print ('et = ' + str(et)[5:10] + ' seconds')
winsound.Beep(2000, 250) # audible signal; parameters are hz, ms
print ("Switch valve to bypass position")

# if using remote controlled solenoid valve include following lines
'''
# switch valve to bypass position
try:
     serRelay = serial.Serial(RelayPort, baud, timeout = 1)
     print("Relay comm port " + RelayPort + ' open')
```

```
            serRelay.setDTR(False) # turn on pin 4 of DB9 serial connector
        except:
            print('unable to open Relay Comm Port ' + RelayPort)
            return
        '''

        # store sample info to log file
        logcsv = cpath + infile.rstrip('.csv') + '_' + 'output' + '.csv'
        flag = exists(logcsv)
        with open(logcsv, 'w', encoding='UTF8') as f:
            writer = csv.writer(f)
            if not flag:
                header = ['datetime', 'sampvol(l)', 'pprofile', 'operator']
                # write the header
                writer.writerow(header)
            data = [dt_string, sampvol, infile, operator]
            writer.writerow(data)

    else: # adjust flow to fixed rate
        print()
        print('Setting flow to '+ flpm +' lpm')
        astr = devID + 'S' + flpm + '\r'
        time.sleep(0.9)
        SendSerMFC(astr)
        ReadSerMFC()

def Shutdown():
    serMFC.close()
    print('done')
    window.destroy()



# attempt to connect to serial device
try:
```

```
    serMFC = serial.Serial(MFCport, baud, timeout = 1)
    print(MFCport + ' open')
except:
    print('unable to open MFCport ' + MFCport)
    print('shutting down')
    sys.exit()

# create GUI popup window
window.title('flow control')
window.geometry('150x500-50-50')
instructions = tkinter.Label(window, text = 'Select mode')
instructions.pack()
h=7
w = h*3
buttonL = tkinter.Button(window, text='Light',command=lambda f='1':
SetMFC(f),bg='Yellow',height=h,width=w)
buttonS = tkinter.Button(window, text='Stop',command=lambda f='0':
SetMFC(f),bg='Red',height=h,width=w)
buttonP = tkinter.Button(window, text='PuffSeq',command=lambda f='S':
SetMFC(f),bg='Green',height=h,width=w)
buttonQ = tkinter.Button(window, text='Quit',command=lambda :
Shutdown(),bg='Grey',height=h,width=w)
buttonL.pack()
buttonS.pack()
buttonP.pack()
buttonQ.pack()

window.mainloop()
```

References

1. Bhattarai, C., et al., *Physical and Chemical Characterization of Aerosol in Fresh and Aged Emissions from Open Combustion of Biomass Fuels.* Aerosol Science and Technology, 2018. **52**(11): p. 1266-1282.
2. Sengupta, D., et al., *Light Absorption by Polar and Non-Polar Aerosol Compounds from Laboratory Biomass Combustion.* Atmospheric Chemistry and Physics, 2018. **18**(15): p. 10849-10867.
3. Samburova, V., et al., *Polycyclic aromatic hydrocarbons in biomass-burning emissions and their contribution to light absorption and aerosol toxicity.* Science of the Total Environment, 2016. **568**: p. 391-401.
4. Son, Y., et al., *Carbonyls and Carbon Monoxide Emissions from Electronic Cigarettes Affected by Device Type and Use Patterns.* International journal of environmental research and public health, 2020. **17**(8): p. 2767.
5. Khlystov, A. and V. Samburova, *Flavoring compounds dominate toxic aldehyde production during e-cigarette vaping.* Environmental science & technology, 2016. **50**(23): p. 13080-13085.
6. Chow, J.C., et al., *The DRI thermal/optical reflectance carbon analysis system: description, evaluation and applications in US air quality studies.* Atmospheric Environment. Part A. General Topics, 1993. **27**(8): p. 1185-1201.
7. Subramanian, R., A.Y. Khlystov, and A.L. Robinson, *Effect of peak inert-mode temperature on elemental carbon measured using thermal-optical analysis.* Aerosol Science and Technology, 2006. **40**(10): p. 763-780.
8. Subramanian, R., et al., *Positive and negative artifacts in particulate organic carbon measurements with denuded and undenuded sampler configurations special issue of aerosol science and technology on findings from the fine particulate matter supersites program.* Aerosol Science and Technology, 2004. **38**(S1): p. 27-48.
